# Morphological Diversity, Genetic Characterization, and Phytochemical Assessment of the Cypriot Tomato Germplasm

**DOI:** 10.3390/plants10081698

**Published:** 2021-08-18

**Authors:** Filio Athinodorou, Petros Foukas, Georgios Tsaniklidis, Anastasios Kotsiras, Antonios Chrysargyris, Costas Delis, Angelos C. Kyratzis, Nikolaos Tzortzakis, Nikolaos Nikoloudakis

**Affiliations:** 1 Department of Agricultural Science, Biotechnology and Food Science, Cyprus University of Technology, Limassol 3036, Cyprus; fm.athinodorou@edu.cut.ac.cy (F.A.); petrosfoukas@gmail.com (P.F.); a.chrysargyris@cut.ac.cy (A.C.); nikolaos.tzortzakis@cut.ac.cy (N.T.); 2 Department of Viticulture, Vegetable Crops, Floriculture and Plant Protection, Institute of Olive Tree, Subtropical Crops and Viticulture, Hellenic Agricultural Organization ELGO-DIMITRA, Mesa Katsabas, 71307 Heraklion, Greece; tsaniklidis@elgo.iosv.gr; 3 Department of Agricultural Technology, School of Agricultural Technology and Food Technology and Nutrition, University of Peloponnese, 24100 Kalamata, Greece; akotsiras@teikal.gr (A.K.); delis@us.uop.gr (C.D.); 4 Vegetable Crop Sector, Agricultural Research Institute—Ministry of Agriculture, Rural Development and Environment, Nicosia 1516, Cyprus; A.Kyratzis@ari.gov.cy

**Keywords:** ascorbic acid, carotenoids, heirloom varieties, landraces, microsatellites, minerals, phenolics, *Solanum lycopersicon*

## Abstract

Tomato (*Solanum lycopersicum* L.) is considered one of the most valuable and versatile vegetable crops globally and also serves as a significant model species for fruit developmental biology. Despite its significance, a severe genetic bottleneck and intense selection of genotypes with specific qualitative traits have resulted in the prevalence of a restricted number of (geno)types, also causing a lack of diversity across widespread cultivated types. As a result, the re-emergence of landraces as well as traditional and heirloom varieties is largely acknowledged as a countermeasure to restore phenotypic, phytochemical and genetic diversity while enriching the aroma/taste tomato palette. On those grounds, the Cypriot tomato germplasm was assessed and characterized. Ten landrace accessions were evaluated under greenhouse conditions and data were collected for 24 IPGRI discrete phenotypic traits. Grouping of accessions largely reflected the fruit shape and size; four different fruit types were recorded across accessions (flattened, heart-shaped, rounded and highly rounded). Moreover, a single run panel consisting of ten SSRs was developed and applied in order to genetically characterize 190 Cypriot genotypes and foreign heirloom varieties. Based on genetic indexes it was established that tomato landraces have a rather low level of heterogeneity and genetic variation. Finally, mineral and phytochemical analyses were conducted in order to estimate biochemical attributes (total phenolics, ascorbic acid, lycopene, *β*-carotene, total soluble content, titratable acidity) across genotypes; thus, ascertaining that the Cypriot panel has a high nutritional value. Due to the thermo-drought adaptation and tolerance of these genotypes, the current study serves as a roadmap for future breeding efforts in order to incorporate desirable traits or develop novel tomato lines combining resilience and alimentary value.

## 1. Introduction

Tomato (*Solanum lycopersicum* L.) is one of the most highly appraised and versatile vegetable crops globally and is widely cultivated for fresh-fruit consumption, grown as an industrial/cash crop or even for processed (concentrate, juice, paste, powder, soup or sauce) products [1]. Globally, tomato harvests can yield more than 180 million tons [2] indicating its significance in terms of production across countries. Moreover, tomatoes can be grown either in a field or in greenhouses, while soilless cultivation schemes enjoy increasing popularity. Such crop production systems’ flexibility allows for worldwide productivity, even in marginal or desert areas [3]. From an alimentary supply standpoint, tomato fruits are a significant source of vitamins, fibers, sugars, and essential minerals for the human diet [4]. Moreover, besides its universal farming and financial standing as a crop, tomato is also a pre-eminent model system for fundamental and applied plant genetic studies focusing on the ripening process [5], secondary metabolism pathways [6] and immunity against biotic stress [7,8].

Wild ancestral species are believed to be native to the Pacific shore of South America. Consequently, tomato crop wild relatives (CWRs) are adapted to an assortment of edaphoclimatic environments spanning from the sea level up to the highlands of the Andean sierra, and thrive in arid, brackish water or rainy conditions [1]. Despite the long period of time since tomato plants were introduced into the Old World, domestication schemes and routes are still largely controversial, oscillating among a Peruvian or a Mexican center of origin. Nonetheless, historical evidence dictates that the dissemination of tomato occurred eastwards and was attributed to the conquistadors’ explorations [9].

For that matter, early introduced genotypes (up to the nineteenth century) were mostly open-pollinated and on-farm propagated at small local scales. As a result, novel varieties emerged via spontaneous mutations and/or recombination of pre-existing genetic diversity [10]. The bulked selection mode and the concurrent breeding from multiple farmers resulted in a plethora of tomato types and rich germplasm. Nonetheless, since tomatoes are mainly self-fertilized, intercrosses among different plants were fairly infrequent while seedlings resembled a parental phenotype. This, in turn, resulted in upholding fixed tomato populations delineated as ‘heirlooms’ which are inimitable in their size, shape, colour, taste and aroma [1].

Nowadays, the fruit weight across modern tomato varieties range from about 10 g in acorn and cherry type tomatoes to more than 450 g in some beefsteak fresh tomatoes [11]. To this regard, it has been established that tomato CWRs present a diverse array of characteristic small-size fruits [12]; which nonetheless have prominent levels of valuable nutritional elements like lycopene, and elevated soluble solid content (SSC) [13]. Still, the deliberate selection of genotypes, domestication and intensive breeding aiming at the enhancement of fruit size and durability, resulted in alternating fruit characteristics and diversification of fruit weight, shape and color [14,15]. Regrettably, the demand from markets for big, homogenous fruits with long postharvest life promoted the production of varieties and hybrids with such characteristics, causing a genetic bottleneck in terms of variability. Such alterations resulted in a general downgrade of nutritional properties, which are presently somewhat inferior in modern cultivars and hybrids mostly due to the increase of tomato fruit size and intensive cultivation crop schemes [16]. Moreover, the selection of a restricted number of genotypes has caused a genetic bottleneck in terms of variability, and currently less than 10% of the total genetic diversity is present in the *S. lycopersicum* gene pool [15,17,18]. As a result, there is a vivid debate among consumers on the usage of recently established commercial varieties at the expense of heirloom cultivars, since the latter are considered superior in several qualitative aspects regarding flavor, aroma and phytochemicals [4].

Currently, a significant turn towards qualitative nutriment attributes is occurring. Moreover, food science and technology have been shifting away from merely the notion of adequate calorie intake, towards an optimal and efficient nutrition scheme. Thus, currently, the breeding aim is to promote the consumption of bio-functional foods and contribute to improved physical health, therefore preventing the risk of diseases [19]. Tomatoes are characterized by a high nutritious value and furthermore contain an extensive assortment of natural antioxidants, such as ascorbic acid, carotenoids, flavonoids and phenolic substances [20]. The total phenolic ratios have been generally related to reactive oxygen species (ROS) scavenging and therefore are believed to be protective against cellular oxidative damage; by extension, they can be beneficial against severe illness like cardiovascular diseases, development of cancer or even neurodegenerative disorders [21].

Tomato landraces epitomize the most basic form of existing cultivars and are largely regarded as intermediates of the breeding process [22]. Opposed to modern-day cultivars, landraces are very heterogeneous since they were recurrently designated for their performance in adverse and low-input agricultural environments, as well as qualitative criteria e.g., aroma [23]. Due to a unique combination of taste, tradition and functionality, heirlooms and tomato landraces are in the breeders’ spotlight and are at the epicenter of breeding efforts to re-establish nutrient and flavorsome tomato fruits. As a result, studies aiming to properly characterize the tomato germplasm are increasingly gaining attention.

When aiming to harness the phenotypic and phytochemical variation of cultivated tomato, and in order to engage this assortment in applied and basic breeding schemes, it is vital to quantify and count such traits in a precise and factual manner. Moreover, phenotypic and biochemical analyses of genetic resources are essential in order to appraise the genetic basis that connects these characters and their overall yields [24]. However, the evaluation of such traits can be challenging and laborious, mostly due to the number of such traits [25], and should be accompanied by genetic markers that are not affected by growing conditions. Nonetheless, while the majority of scholars emphasize the assessment of modern, as well as traditional cultivars, studies at the regional level are largely uncommon. It is generally thought that indications regarding the disparities within tomato landrace germplasm are still rather inadequate, since regionally grown traditional genotypes should not be conceived as strictly homogenous [26].

In view of these remarks and due to the shortage of comprehensive information regarding the Cypriot tomato genetic recourses, the objectives of the current study were: (i) to perform a morphological description using scores and descriptors according to worldwide standard norms (International Plant Genetic Resources Institute (IPGRI) descriptors); (ii) to use microsatellite genetic markers and append landraces of other origins to define the genetic structure and intra-relations of Cypriot tomato varieties; (iii) to assess the variation in phytochemicals and other traits of tomato fruits from plants grown under uniform greenhouse conditions and (iv) to investigate the relations between them. The overall goal was to increase the information regarding the local tomato germplasm, which has the potential for exploitation in modern cultivation schemes and direct usage in marketplaces, breeding schemes and for future genomic surveys.

## 2. Materials and Methods

### 2.1. Plant Material

The plant material used in the current study consisted of 19 discrete landraces. Ten were Cypriot landraces, namely AR100731, ARI00732, ARI00733, ARI00734, ARI00735, ARI00736, ARI00737, ARI00872, ARI00905 and ARI00906 (Table 1) reserved at the genebank of the Agricultural Research Institute (ARI), Cyprus. These accessions were studied in terms of morphological traits and biochemical properties, and were genotyped with microsatellites. Furthermore, eight Greek and one French heirloom varieties were genotyped in order to detect possible inter-genetic affiliations to the Cypriot tomato germplasm.

### 2.2. Cultivation Scheme

All Cypriot tomato genotypes were seeded at a nursery (using peat as a substrate) and transplanted at a two-leaves-stage to a greenhouse (March 2018), according to a randomized complete block design, and within row distance of 50 cm. Three discrete blocks were employed and within each plot five plants per accession were studied (in total 150 were evaluated). For fruit morphological traits, at least ten randomly selected fruits from the second and third truss were analyzed as indicated (IPGRI descriptors for Tomato). Irrigation was provided via drippers at a frequency of two days (for 30 min). Five intervals of fertilization were conducted using an all-purpose water-soluble commercial fertilizer (20-20-20), while pest management against whiteflies and *Tuta absoluta* was conducted via the repeated application of insecticides (Nuprid 200 SL and Bolivar 1.8 EC, respectively) when needed, before anthesis.

### 2.3. Analysis of Phenotypic Data

On a single tomato plant basis, 24 discrete agro-morphological traits were scored as detailed in Supplementary Table S1. In brief, the recorded IPGRI descriptors were categorized as vegetative (6 traits), inflorescence (4 traits), fruit descriptors (12 traits) and agronomic characteristics (2 traits) and corresponded to scale, ordinal and nominal data types. Since such traits are a mixture of numerical and categorical variables, several statistical incongruities can occur when applying standard component analyses; hence for the evaluation of morphological descriptors, an R package dedicated to multivariate analysis of mixed data (PCAMIX) was employed [27]. Euclidean distances were calculated across landraces and a distance matrix was produced. Moreover, a principal component analysis (PCA) plot was constructed from the combined morphological descriptors to depict the affiliations across the tomato landraces, and squared loadings were determined in order to detect correlations among the trait descriptors.

### 2.4. DNA Extraction

For nucleic acid extraction, tips from young tomato leaves were collected and kept among moist towel paper on ice, till storage at −70 °C. DNA extraction was conducted using the Dneasy Plant Mini Kit (Qiagen, Hilden, Germany) according to the manufacturer’s instructions. The purity and concentration of DNA were estimated by means of nanodrop spectrophotometry. Ten plants per accession were randomly sampled.

### 2.5. PCR and SSR Genotyping

Fifteen simple sequence repeat (SSR) markers were initially screened for the preliminary analysis of the Cypriot tomato germplasm. Based on the efficiency, reproducibility and allele size generated, ten loci (Supplementary Table S2) were selected for the full panel of genotypes. All forward primers used were designed to have a universal M13(-21) sequence at the 5′-end and were extended by an overlapping M13 labeled primer (FAM^TM^, JOE^TM^ ROX^TM^, or TAMRA^TM^). This permitted a one-tube, single-reaction nested PCR, as previously described [28]. Based on the fragment sizes from pilot reactions, four multiplex panels (one per fluorescent dye) were determined.

For PCR reactions, each panel master mix contained 50 ng of template DNA, 10 pmole of the labeled M13 tailed forward primer, 10 pmole of the reverse and 2.5 pmole of the forward primer, 0.2 mM dNTPs, 0.5 U KAPA Taq DNA Polymerase (Kapa Biosystems, Basel, Switzerland) and a 2.5 mM final concentration of MgCl_2_ in a 12 μL final reaction volume. Conditions for the PCR amplification were: 94 °C (5 min for initial denaturation), followed by 39 cycles at 94/56/72 °C (60 s), and a final extension at 72 °C for 30 min.

Amplification products were verified using a standard 2% agarose electrophoresis and diluted at a 1:40 ratio with dd H_2_O. Diluted PCR products across panels were all mixed in one tube (per accession). One μL of the mixture was added to 10 μL deionized formamide and 0.2 μL of DNA size standard (GeneScan 500-LIZ, Applied Biosystems, Foster City, CA, USA), before denaturing at 95 °C (5 min). Allele fragments were separated by capillary electrophoresis using an Applied Biosystems 3130^®^ Genetic Analyzer (Applied Biosystems, Foster City, CA, USA).

### 2.6. Molecular Data Analysis

Allele fragments were identified (de-multiplexed) by means of fluorochrome colour and/or bin size, and tandem software was utilized to verify/correct bins [29]. Microsatellite data curation and formatting was performed via the MS Excel add-in GENALEX v. 6.501 [30]. All genotypes were included for calculating allelic frequencies across loci studied. In order to assess the discriminating power among unique genetic profiles, a genotype accumulation curve was constructed. Additionally, genotypic diversity was assessed with several indexes (H: Shannon–Wiener Index of Multi-locus Genotype (MLG) diversity, G: Stoddart and Taylor’s index of MLG diversity, lambda Simpson’s index, E.5: Evenness of the alleles and Hexp: Nei’s unbiased gene diversity). In order to determine allelic abundance, several indexes were evaluated: Na = No. of Different Alleles; Ne = No. of Effective Alleles = 1/(Sum pi^^2^); I = Shannon’s Information Index = −1* Sum (pi * Ln (pi)); Ho = Observed Heterozygosity = No. of Hets/N; He = Expected Heterozygosity = 1 − Sum pi^^2^; uHe = Unbiased Expected Heterozygosity = (2N/(2N − 1)) * He; F = Fixation Index = (He − Ho)/He = 1 − (Ho/He), where pi is the frequency of the i^th^ allele for the population and sum pi^^2^ is the sum of the squared population allele frequencies. Moreover, the proportion of polymorphic loci (PIC) and discrimination power (Dp) of each locus was determined.

The same dataset was similarly used to test for linkage disequilibrium and Hardy–Weinberg equilibrium (HWE) in the tomato accessions. Genetic relationships between individuals (MLGs) were assessed using the ‘dissimilarity’ distance algorithm and visualized as a minimum spanning network (MSN) and a discriminant analysis of principal components (DAPC). All of the above-mentioned statistics/analyses were performed using the Poppr (V. 2.8.5) package [31] and the RStudio suite (V 1.2.5033; R V 3.6.2). The PIC and Dp indexes were calculated using the iMEC: Online Marker Efficiency Calculator (https://irscope.shinyapps.io/iMEC/ (accessed on 1 August 2021)).

A phylogenetic tree was also constructed using the binary template (converted from allele size) using the R package polysat [32]. An approximate likelihood-ratio test (aLRT) for branch support was achieved by means of the SH-like parameter as previously described [33]. The Newick-formatted tree was displayed and manipulated using the iTOL v4 server [34].

A Bayesian statistic-employing method for estimating genetic kinship was performed using Structure 2.3.4 [35]. The admixture model was selected and 10 independent repeats per K value (extending from 1 to 20) were run. Each run involved 100,000 iterations of the burning period and 500,000 post-burning simulations. Validation of the most probable number of K-clusters and visualization was achieved using the Clumpak server (http://clumpak.tau.ac.il/).

In order to examine the possible correlation of mean morphological traits with the genetic distance of landraces, a mantel test (999 permutations) was computed using the distances matrix of morphological descriptors and the genetic distances matrix calculated via the GENALEX v. 6.501 software [30].

### 2.7. Tomato Fruits Sample Preparation

Harvesting (approximately 30 to 40 days after anthesis) was performed at the same ripeness level (red ripe; more than 90% of the surface had red colour) across all tomato fruits from the second and third trusses. Tomatoes were washed with dH_2_O, dried with absorbent paper and immediately placed at −80 °C to stop possible metabolic processes. For the phytochemical analyses, fruits were cut in quarters and at least five discrete fruits were bulked in order to create one sample (biological replicate). Bulked samples were blended and juice was lyophilized. In total, three discrete biological repetitions were estimated across phytochemical analyses.

### 2.8. Quantification of Total Phenols (TP)

Total phenols were estimated following the Folin–Ciocalteu reagent methodology revised for a microplate reader, as previously reported [36]. To attain tomato extracts, one mL of absolute ethanol was added to 0.05 g of lyophilized tomato-fruit powder. Samples were sonicated for 10 min and centrifuged at 13,000 rpm for five min at room temperature. Forty μL aliquots of extract (supernatant) were added to an equal volume of 0.1 M Folin–Ciocalteau reagent and incubated for five min at constant stirring. Forty μL of 0.5% Na_2_CO_3_ were further added, incubated at 40 °C for 30 min, agitated for one min, and absorbance was estimated at a 750 nm wavelength (Tecan Infinite 200 PRO, Männedorf, Switzerland). For the standard curve, serial dilutions of gallic acid (GA) were employed, and results were expressed as mg GA equivalents/100 g FW. All trials were performed in biological triplicates.

### 2.9. Vitamin C Assessment

Determination of the vitamin C content followed the reduction of the 2,6-dichloroindophenol sodium salt (DCIP) method revised for a microplate reader according to Ochoa-Velasco and co-workers [36]. In general, 0.1 g of lyophilized tomato fruit samples were mixed with 0.1% oxalic acid, incubated for five min at room temperature and centrifuged at 13,000 rpm. Forty μL of extracts were transferred into a 96-well microplate (Eppendorf, Hamburg, Germany). Equal volumes of acetate buffer and 2,6-dichloroindophenol solution (30 mg/L) were added and mixed for one min. Absorbance was estimated at a 515 nm wavelength. For the standard curve, serial dilutions of ascorbic acid (AA) were employed and results were expressed as mg AA/100 g FW. All trials were performed in biological triplicates.

### 2.10. Lycopene and β-Carotene Quantification

Quantification of lycopene and *β*-carotene was conducted according to a method previously described [37]. All pigments were extracted from 0.05 g of lyophilized tomato-fruits powder using as a solvent 10 mL of a 4:6 (*v*/*v*) acetone-hexane mixture. Samples were vigorously vortexed and incubated in dark for 20 min at 4 °C. A five min centrifugation followed and 200 μL of extracts were transferred to a 96-well polypropylene microplate (resistant to organic solvents). Absorbance readings were performed at the following wavelengths: 663 nm, 645 nm, 505 nm and 453 nm, and pigments determination was calculated using the referred equations:

Lycopene (mg/100 mL) = −0.0458 × A_663_ + 0.204 × A_645_ + 0.372 × A_505_ − 0.0806 × A_453_ (1)

*β*-Carotene (mg/100 mL) = 0.216 × A_663_ − 1.22 × A_645_ − 0.304 × A_505_ + 0.452 × A_453_ (2)

All trials were performed in biological triplicates.

### 2.11. Total Soluble Solids (TSS), pH and Titratable Acidity

The juice from three biological replicates was used to estimate the total soluble solids (TSS) via a portable digital refractometer (DR103L, Sun Instruments Corp., Torrance, CA, USA), and results were expressed in Brix. pH was measured with a benchtop pH-meter (Hanna, Woonsocket, RI, USA). Titratable acidity (TA) was assessed employing the potentiometric titration of 0.1 mol/L NaOH up to pH 8.1, using five mL of undiluted juice. Estimations were performed on a DL22 Mettler Toledo titrator (Mettler–Toledo, Inc., Columbus, OH, USA) and were expressed as citric acid g in 1 L of tomato juice.

### 2.12. Macro and Micro Nutrient Content in Tomato Fruits

Tissues were ground into a fine powder and passed through a 30-mesh screen. Each sample (0.5 g) was dry-ashed in a muffle furnace at 515 °C for 5 h. Then, the ash was digested in 3 mL of 6 N HCl and diluted with double-distilled water up to 50 mL. The concentrations of P, K, Ca, Mg, Mn, Zn and Cu were determined by ICP (Perkin Elmer-Optical Emission Spectrometer, OPTIMA 2100 DV, Waltham, MA, USA). Nitrogen was determined by the Kjeldahl method (BUCHI, digest automat K-439 and Distillation Kjelflex K-360, Switzerland).

### 2.13. Generalized Procrustes Analysis (GPA)

In order to combine morphological, genetic, mineral and phytochemical analyses, we employed a Generalized Procrustes Analysis (GPA) employing all quantitative traits. The FactoMineR, an R package dedicated to multivariate Exploratory Data Analysis was used under the ‘Commandeur’ algorithm (http://factominer.free.fr/).

## 3. Results

### 3.1. Morpho-Agronomical Variation across Cypriot Tomato Landraces

**Figure 1:**
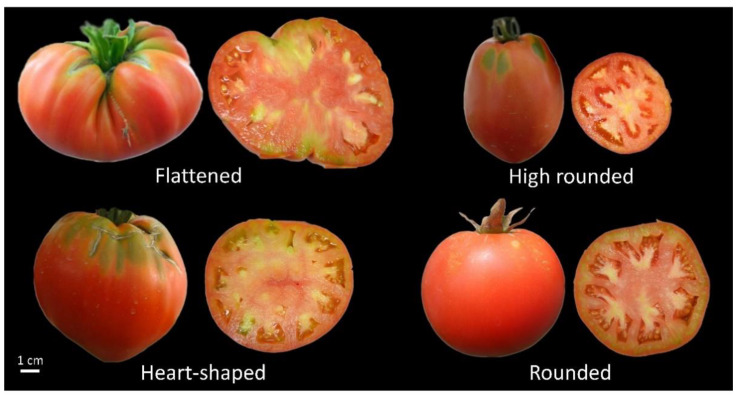
Examples of fruit types (red ripe stage) in the Cypriot tomato landraces panel.

In total, 24 discrete IPGRI descriptors were employed (Supplementary Table S1) and measured in order to assign tomato genotypes to morphological and agronomic clusters (Supplementary Table S3). Ten characters were nominal, eight ordinal and six scale descriptors. Across the Cypriot tomato landraces four characters were found to be uniform (plant growth type, leaf type, corolla colour and style hairiness) and thus further discarded from the analyses. Most of the accessions had a red exterior colour at the red ripe stage; four accessions (ARI00733, ARI00734, ARI00872 and ARI00906) were found to differ and had a soft pink hue. All genotypes could be categorized into four predominant fruit shapes according to the IPGRI criteria (Figure 1). Accessions ARI00731, ARI00732, ARI00737 and ARI00872 had a flattened shape (beefsteak tomato), while landraces ARI00733, ARI00905 and ARI00906 had equally large fruits; but a heart-shaped figure. ARI00735 and ARI00736 were highly similar across traits and had a typical round fruit shape. Accession ARI00734 had the smallest fruit size compared to others and presented a saladette shape (highly rounded). Most of the accessions presented green shoulders except accessions ARI00735 and ARI00736 where the intensity of that trait was significantly lower (Figure 1). The latter alongside landrace ARI00734 were also distinctively different according to the style shape since were the only to present a simple instead of a fasciated shape. Moreover, these two tomato types had a highly exerted style position.

All quantitative fruit traits had statistically significant differences across landraces and a probability ranging from *p* = 0.014 (number of days to flowering) to *p* < 2.2 × 10^−16^ (number of locules). The number of cavities containing seeds was largely correlated to fruit size and ranged from 5.80 ± 1.03 locules for accession ARI00734 to 13.73 ± 2.46 locules for landrace ARI00906 (Supplementary Figure S1). Additionally, a wide range of variation was established for these characters; the coefficient of variation (CV) was calculated as the ratio of the standard deviation to mean values (Supplementary Table S3). Across traits the highest CV (34.63%) was established for fruit weight. Indeed, fruit weight was a character that largely variated; since in the Cypriot tomato collection there were medium-size fruit genotypes (211.20 ± 75.73 g for ARI00735) and exceptionally large ones (530.00 ± 104.34 g for ARI00737). Pearsons’ correlations, depicted as a heatmap, revealed that several traits were significantly affiliated (Supplementary Figure S2). Fruit weight and width were the two characters that were highly positively associated (r = 0.953, *p* < 0.01; Sig. 2-tailed), while the number of locules was also correlated to fruit width (r = 0.720, *p* < 0.05; Sig. 2-tailed) and weight (r = 0.743, *p* < 0.05; Sig. 2-tailed).

**Figure 2:**
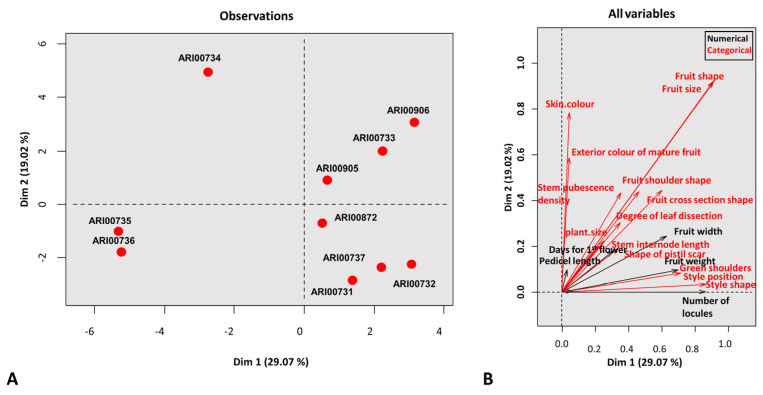
Principal component analysis on the two first eigenfactors based on agro-morphological descriptors (**A**). Categorical and quantitative variables explaining morphological variance across the Cypriot tomato genotypes (**B**).

A principal component analysis (PCA) was plotted (Figure 2) aiming to depict an overall outline of the structural variation across landraces. A multivariate analysis procedure dedicated for mixed data as described in the PCAMIX R Package was followed. The first two components employed explained an accumulated 48.09% of the variation, a relatively moderate value possibly due to the high number of traits analyzed and the presence of both quantitative and qualitative traits. A heavy weight on the discrimination capacity was obtained for nominal and ordinal characters and in particular for fruit shape and size (which were highly affiliated). Regarding the quantitative traits, a significant weight was attributed to the number of locules and fruit width. Specifically, clustering was primarily affected by the small fruit, highly rounded and angular cross section characters (Supplementary Figure S3). The graphical illustration of the PCA presented a broad diffusion of tomato landraces. The first dimension that explained approximately 30% of total variability was adequate to differentiate round and highly rounded (smaller fruit) landraces from the core of the collection. Larger fruit genotypes were further demarcated on the second axis to heart-shaped and flattened fruit accessions. Nonetheless, a close intergroup affiliation was clear. From an agro-morphological standpoint, accessions ARI00735 and ARI00736 were found to be highly similar and did not present significant inter-variations.

### 3.2. DNA Fingerprinting, Diversity Indexes and Genetic Relationship across the Tomato Germplasm

The Cypriot tomato germplasm collection (ten accessions) was genotyped alongside nine foreign heirloom tomato cultivars. After preliminary experiments where fifteen primer sets were screened, ten loci were selected in terms of fragment size bins, allele variability and reproducibility of proliferation (Supplementary Figure S4). The genotype accumulation curve (Supplementary Figure S5) shows that the 10 SSR loci were adequate to define all the multi-locus genotypes (MLGs) present across landraces. In total, 33 distinct genotypes and 32 discrete alleles were found from a pool of 190 individuals (Table 2 and Table 3). The probability of identity (PI) of two samples having an identical genotype was also computed for the dataset, and it was estimated that the collective capacity of the ten SSR loci resulted in an average PI value of 8.8 × 10^−2^ across populations. All loci were found to be polymorphic across landraces, still, the percentage of polymorphic loci within the landraces and heirloom varieties was significantly reduced. Since tomatoes are highly autogamous plants and have been present for a short period in the Old World, a restricted amount of genetic and allelic diversity is expected within landraces. Across loci, two to four different alleles were detected having a mean value of 3.2 (Table 3).

Moreover, the observed and expected heterozygosity indexes were also restricted in range and in all cases were below the 0.5 threshold; showing a restricted allelic variation among and within landraces. Cypriot landraces were found to be virtually entirely homogeneous, since all individuals within a landrace (except ARI00735 and ARI00736) presented a single genotype. Nonetheless, across loci and genotypes a moderate amount of genetic diversity was evident based on several diversity indexes (Supplementary Figure S6; Table 3). Fixation indexes (F) were mostly negative and at the lowest point (−1), suggesting an excess of heterozygosity, due to negative assortative mating, or selection for heterozygotes. Moreover, we explored the probability that loci were under HW equilibrium (Supplementary Figure S7). Ιt was established that several loci were in HWE (*p* < 0.5), except loci LEMDDNA, LELEUZIP and EST258529, where a significant (*p* = 0.05) value of disequilibrium was established. This suggests that sexual propagation has also occurred in the lineage of Cypriot tomato varieties, even though at a reduced rate.

The analysis of molecular variance (AMOVA) revealed that 58% of the total variation was attributed to the genetic diversity among varieties, while the remaining 42% was attributed to the genetic variability within landraces and heirloom varieties. F_st_ values (Supplementary Table S4) across groups were significant at the *p* < 0.001 level and ranged from 0 (among Cypriot landraces ARI00733 and ARI00906 that were grouped as one cluster) up to 0.864 (among the ARI00733/ARI00906 group and the Greek heirloom variety ‘Stithos Aphroditis’).

**Figure 3:**
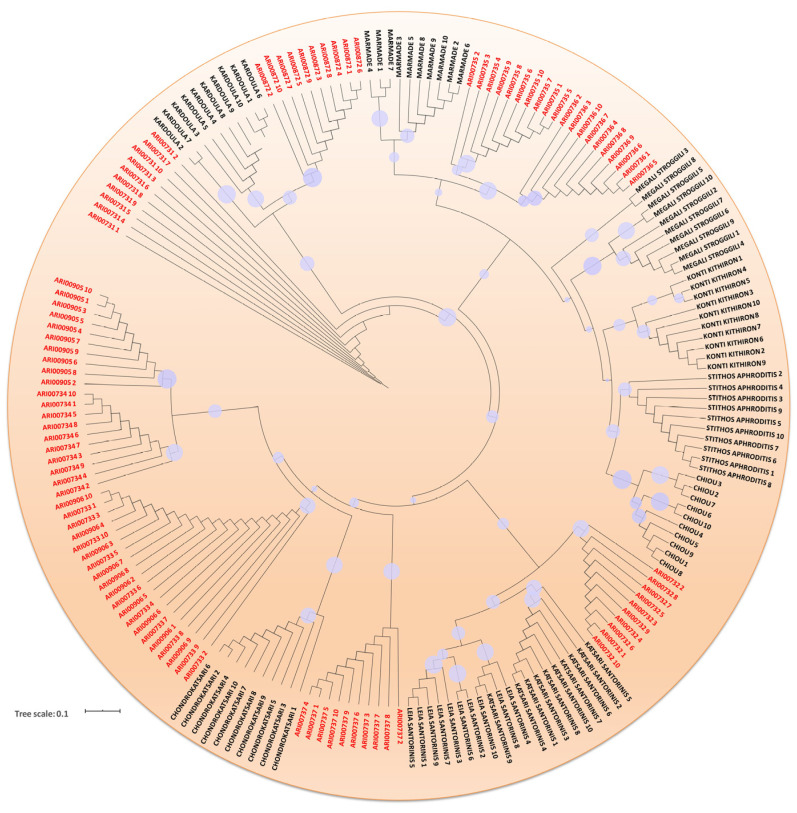
Circular dendrogram depicting genetic affiliations among 190 tomato genotypes. Cypriot accessions are depicted with a red font. The size of circles on nodes correlates to bootstrap support values.

Genetic distances across genotypes were used to construct a dendrogram depicting linear phylogenetic relationships and relevant bootstrap support values (Figure 3). Additionally, a Mantel test using 999 permutations was employed to evaluate possible correlation to morphological characters. A rather weak relation was detected (r = 0.293; *p* < 0.045). It was established that within the tomato germplasm, complex genetic relationships could be identified; but all landraces and heirloom varieties were clearly demarcated. Moreover, bootstrap values further confirmed that these varieties have significant discrepancies that reflect a discrete genetic markup. However, Cypriot accessions ARI00733 and ARI00906 (both having the same fruit morphotype) were found identical and thus could signify a remarkably close genetic ancestry. Accessions ARI00735 and ARI00736 (the only accessions having a typical round shape in the current collection) were also found highly affiliated but presented a clear cut-off value; hence stand as discrete varieties or clones. Grouping of genotypes did not clearly reflect the country of origin nor did it absolutely reflect morphotypes, signifying that the markers selected are not strongly associated to agro-morphological traits but are rather unrelated.

**Figure 4:**
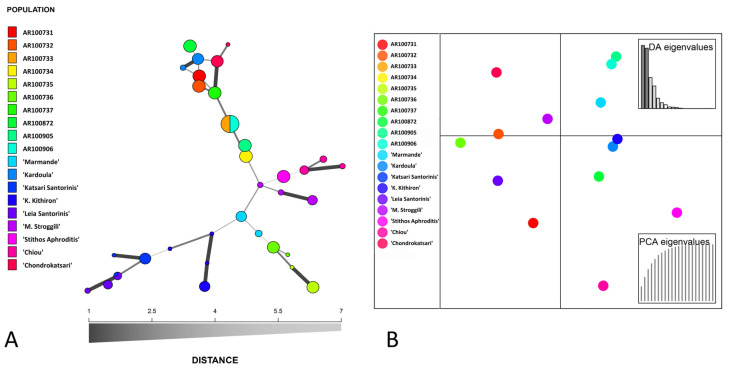
(**A**) Minimum spanning network (MSN) and affiliations of multi-locus genotypes (MLGs) as constructed based on dissimilarity genetic distances. Linear and reticulated connections are evident across genotypes (**B**). Discriminant analysis of principal components (DAPC) among Cypriot landraces and Greek heirloom tomato varieties.

A minimum spanning network (MSN) was also constructed (Figure 4A) to detect possible reticulate genetic relationships. Additionally, a discriminant analysis of principal components (DAPC) concerning the identification and designation of clusters with genetically associated individuals was employed (Figure 4B). These analyses also confirmed that there is not any plain structure based on geographical proximity, but that an interchange of genetic material must have taken place. Moreover, it was clear that intra-genetic discrepancies were comparably restricted and did not significantly affect the clustering, since all individuals within a landrace or heirloom variety were highly affiliated.

**Figure 5:**
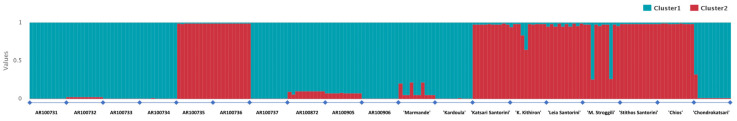
Structure analysis for Cypriot tomato germplasm depicting probability for genetic cluster assignment at K = 2.

A Bayesian-based method was additionally employed in order to survey the distribution of the genetic diversity and the population structure of the tomato germplasm (Figure 5). The optimal value for the ad hoc test, based on the second order rate of change of the likelihood function with respect to ΔK, was detected for K = 2 (ΔK = 5615). Many individuals had a percentage of membership larger than 0.8; nonetheless, the Bayesian inference discovered considerable admixtures within landraces. Cypriot landraces were mostly grouped within the first cluster apart from ARI00735 and ARI00736 that had significant genetic differences to the core of Cypriot germplasm. Interestingly, the Greek heirloom varieties ‘Kardoula’ (that means ‘heart-shaped’ in Greek) and ‘Chondrokatsari’ (that translates ‘fat and ribbed’ in Greek), were also highly affiliated to the Cypriot genetic pool.

### 3.3. Physicochemical Characterization of Cypriot Tomato Landraces

#### 3.3.1. Mineral Composition

Macro- and micro-elements determined for the Cypriot tomato landraces revealed that statistically significant differences (*p* < 0.01) exist across the tomato genotypes (Table 4). Average values of mineral levels confirmed that Cypriot tomatoes are a rich source of vital elements; regardless of the fruit type. Nonetheless, in several cases, it was established that a specific fruit type corresponded to elevated mineral amounts. Rounded tomatoes (ARI00735 and ARI00736) seem to contain higher levels of phosphorous and calcium (macro elements), as well as micronutrients (Zn, Mn and Cu), compared to other types (Table 4). On the other hand, heart-shaped and flattened tomato types were found to be comparable under the prism of mineral nutrient value, since ANOVA clustering did not reveal significant departures across macro- and micro-elements. Still, coefficients of variance generally revealed that the accumulation of minerals in the fruits of tomato is a complex trait with significant deviations; CV percentage ranged from 6.55% (N) to 43.13% (Ca).

#### 3.3.2. Tomato Fruit Qualitative Characteristics

The evaluation of fruit qualitative characteristics pertained to the following traits: pH, acidity (% citric acid), total soluble solids (Brix), ascorbic acid (vitamin C), total phenolics, lycopene and *β*-carotene levels (Table 5). Statistically significant differences were confirmed for all traits across Cypriot tomato landraces. In general, the water content of tomato fruits ranged from 92.87% (ARI00732) to 96.57% (ARI00736) with an average of 94.53%. Moreover, tomato fruit water percentage had a strong negative correlation to the number of locules (r = 0.81; *p* < 0.05). Interestingly, a mild negative correlation (r = −0.63; *p* < 0.05) among the pH and titratable acidity (calculated as citric acid) was estimated, indicating that several organic acids (including malic acid) make up the acidic profile of Cypriot tomato landraces and that the total titratable acidity is a much more complex trait than pH. Moreover, pH discrepancies across genotypes were smaller—in absolute numbers—comparative to TA; an element that also reflects the vast difference in the ANOVA probability value across groups (*p* = 0.00018 for pH/*p* = 2.2 × 10^−12^ for TA; Table 5).

Significant differences in sugar levels were also recorded via the estimation of the refractive index, the total soluble content (TSS) solids concentration and the TSS/TA ratio. On average, approximately 4.11 °Brix were estimated for Cypriot landraces, making the Cypriot germplasm neither sweet nor sour. Specifically, landrace ARI00735 had the lowest TSS content (3.2 °Brix), while ARI00737 was found to have an increased TSS/TA ratio and almost twice the sugar content (5.07 °Brix).

Cypriot tomato landraces were also found to have substantial levels of ascorbic acid (AA). The analysis showed that the vitamin C capacity of Cypriot tomatoes ranged from a minimum of 24.37 ± 1.04 mg AA/100 g FW for ARI00906, to a maximum of 48.02 ± 0.94 mg AA/100 g FW for landrace ARI00735. Overall, the mean value of AA was above 30 mg AA/100 g FW, suggesting that Cypriot varieties are a rich source of vitamin C.

Additionally, tomato fruits were found to hold significant amounts of phenolic substances at relatively elevated levels as determined using the Folin–Ciocalteu assay (about 7 mg of GAE/100 g FW were estimated on average). Interestingly, the ARI00734 accession (the only pomodoro type in the Cypriot collection) was distinctively disassociated from the remaining cluster of genotypes and presented significantly lower levels of phenolics (4.50 ± 0.16 mg of GAE/100 g FW).

Lycopene and *β*-carotene levels were also evaluated for landraces, and it was proven that significant discrepancies exist across genotypes (*p* = 2.2 × 10^−16^ for lycopene; *p* = 0.00037 for *β*-carotene). Lycopene values ranged from 1.42 ± 0.05 mg/100 g FW (ARI00734) to 5.85 ± 0.04 mg/100 g FW (ARI00905). Surprisingly, landraces ARI00734, ARI00735 and ARI00736 were considered within the same ANOVA group, all presenting low levels of pigments. In the case of *β*-carotene, even though there were significant deviations among the landraces, nonetheless, differences were less broad (Table 5). ARI00735 was established as the landrace with the highest *β*-carotene concentration (0.92 ± 0.02 mg/100 g FW), while ARI00733 (0.42 ± 0.07 mg/100 g FW) alongside to ARI00906 (0.49 ± 0.11 mg/100 g FW) had the lowest.

**Figure 6:**
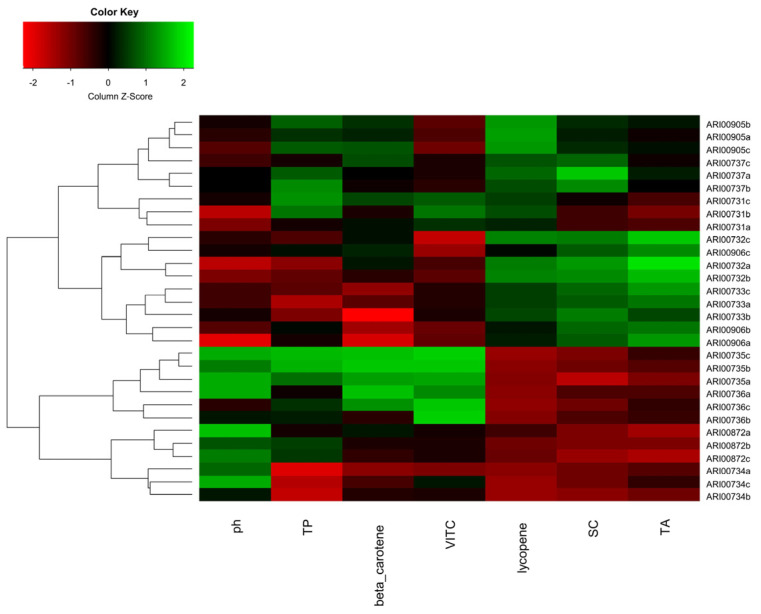
Hierarchical clustering among Cypriot tomato landraces based on phytochemical properties established from three biological replicates.

It appeared that phytochemical grouping did not necessarily correlate to fruit types or morphological traits. Nonetheless, landraces ARI00735 and ARI00736, as well as landraces ARI00733 and ARI00906 were clustered within the same or adjacent ANOVA groups; hence a metabolic affiliation was proven alongside agro-morphological and genetic affinity. To identify the hierarchical phytochemical proximity among the landraces, values of quality traits were standardized, and the transformed matrix was depicted as a heat map (Figure 6). Two major clusters were formed; the first composed of smaller fruited landraces (ARI00734, ARI00735 and ARI00736); while landraces with larger fruits (heart-shaped; ARI00733, ARI00905, ARI00906 and beefsteak; AR100731, ARI00732, ARI00737, ARI00872) were affiliated and formed the second group.

#### 3.3.3. Generalized Procrustes Analysis (GPA)

**Figure 7:**
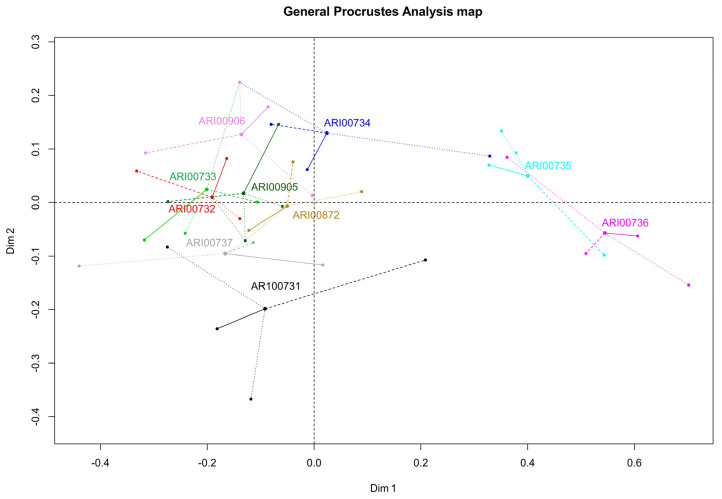
Two-dimensional plot inferred from the combined generalized Procrustes analysis.

In order to combine all types of diverse analyses (morphological traits, allelic data, mineral content and phytochemical properties) into a single analysis, a generalized Procrustes analysis (GPA) was employed (Figure 7). Accessions ARI00735 and ARI00736 (round fruit varieties) were clearly demarcated from the core of the germplasm collection on the first dimension, supporting the overall distinct nature of these landraces. Moreover, the only high rounded variety (ARI00734) had a lower affinity to heart-shaped and flattened-shaped tomatoes. The second axis also placed landraces according to fruit type (all heart-shaped landraces were placed at a positive scale while flattened accessions were mostly at the negative scale), although differences were not as extensive as in the case of rounded cultivars.

Mean values and relative standard deviations across phytochemical analyses. Different letters (a–g) refer to statistically significant differences at *p* < 0.05 as analyzed by one-way ANOVA and the Duncan Multiple Range Test. Coefficient of variation (CV) was calculated as the ratio of the standard deviation to mean values.

## 4. Discussion

Cyprus, the largest island in the eastern Mediterranean basin is located at the crossroad point of three continents (Europe, Asia and Africa) and historically it has been on the map of millenia-old trade routes. Many discrete civilizations have shaped the Cypriot agricultural tradition throughout the centuries, each contributing to the introduction of non-indigenous species; such as tomato. Moreover, Cyprus has a complex edaphoclimatic background that is divided into four discrete geological zones: (a) the Pentadaktylos (Keryneia) zone, (b) the Troodos Ophiolite, (c) the Mamonia zone and (d) the zone of the autochthonous sedimentary rocks [38]. Complimentary to soil type diversity, the agricultural zones of Cyprus range from the sea level to the highlands of the Troodos Sierra (1952 m). In general, conditions across Cyprus are very harsh since during summertime (tomato growing season) temperatures can easily pass and remain above the 40 °C threshold, while soil is mainly characterized as poor, alkalic and rich in calcium. Taken together, the Cypriot germplasm holds a unique global place and the landraces acclimated in such distinct conditions are of fundamental importance and factually represent the Cypriot local identity. Moreover, such lines possibly have a breeding value (irrespectively of their nutrient value) since they could be crossed with more intensive varieties.

Nowadays, a gradual detachment of consumers from established tomato cultivars towards locally-grown traditional cultivars is ongoing. This attitude is revitalizing the interest in heirloom and rustic tomato landraces and is leading to the rediscovery of local agricultural traditions [19]. Hence, several studies are increasingly focusing on the description of agro-morphological traits, diversity analysis and phytochemical characterization of local and antique tomato germplasm [26,39,40,41,42,43,44,45]. In the current study, ten accessions of Cypriot tomato landraces were characterized using 24 agro-morphological descriptors, microsatellite genetic markers (across ten loci) as well as several phytochemical parameters in order to characterize, for the first time in literature, the local tomato germplasm and further evaluate its breeding potential.

### 4.1. Morphological Parameters

All landraces presented an intermediate growth trait and had dense foliage as frequently reported across Mediterranean genotypes [46,47]. As a result, these genotypes are not determinate and thus require pruning, formation and support, in order to sustain fruit production and achieve the optimal nutritional equilibrium. Across landraces, a rich diversity was established at almost all morphological traits. Nonetheless, the intra-variability of Cypriot accessions was lower and genotypes were highly uniform; at least in the cases of ordinal and nominal traits. Still, the coefficient of variation (CV) indexes across quantitative parameters for the collection were elevated (Supplementary Table S3); stressing the diversity of the panel and the possibility of selecting preferable characteristics in future breeding schemes. The heterogeneity of the Cypriot panel is in accordance with similar studies focusing on the phenotypic diversity of Greek [47], Italian [42] and Spanish tomato landraces [43]; although Cypriot genotypes did not reveal distinct intra-varietal morphotypes as previously reported [39].

The present collection included genotypes producing fruits mostly appropriate for local trading and self-consumption, since tomato seeds were initially collected directly from farmers who grew tomatoes for such purposes, rather than having long shelf life for wider distribution, thus, the predominant fruit shapes and colour hues mostly reflected local preferences. We also found a considerable level of variation for fruit size and weight, while in several cases, the fruits exceeded the 500 g threshold, having an extremely large size (Supplementary Table S3). Parisi and coworkers also reported size variation and several morphotypes in the ‘Sorrento’ tomato Italian landrace [44] while Terzopoulos et al. reported that approximately 70% of the Greek tomato landraces studied had fruits with low weight [47]. Such outstanding levels of measured traits have been frequently attributed to both the unique genotypic potential, as well as the environmental adaptability and the capacity of system production [46]. Among morphological traits, the fruit shape and size, ribbing and colour are essential criteria for the definition of a tomato type [48]. Four major types were distinguished across Cypriot landraces (Figure 1). Large fruiting tomatoes having a flattened (ARI00731, ARI00732, ARI00737 and ARI00872) or heart-shaped outline (ARI00733, ARI00905 and ARI00906) were predominate, while two accessions had a more typical rounded figure (ARI00735 and ARI00736). Only one highly rounded type was asserted (ARI00734). In a recent study [44], the analysis of morphological traits in Italian landraces divided the germplasm in two distinctive major groups (flattened, obcordate or oblate fruit shaped and heart-shaped or circular fruits showing angular or circular shape in cross-sections.

The majority of accessions had also distinguished greenback (green shoulders). The shape of the pistil scar was also a significant breeding trait that showed variation across genotypes. In general, flattened/heart-shaped tomato fruits presented an irregular shape of pistil scar (Supplementary Table S3) with the exception of ARI00732, which generally developed a small and round-shaped scar. As previously reported [49], a large and irregular size of the pistil scar can reduce the commercial value of the tomato fruit and the post-harvest shelf-life due to increased water loss and pathogen susceptibility. Hence it can be recognized that promising traits can be found within the Cypriot collection.

All morphological traits were converted to eigenvalues and a PCA was constructed (Figure 2). The PCA analysis of mixed type data revealed that the Cypriot panel was mainly classified into three clusters while one accession remained unrelated (ARI00734). The first two axes explained less than 50% of the total variation. The rather low contribution for each eigenvalue when explaining variation in tomato landraces has been attributed to either a high intra-population variation across genotypes or due to low inter-genetic variation [46]. Additionally, the close geographical proximity (Cyprus has an area of less than 9500 km^2^) may cause a geographical bias that further restricts landrace diversity and narrows the genetic basis.

### 4.2. Genetic Variation

Several molecular marker systems have been employed to survey the genetic diversity of tomato collections such as amplified fragment length polymorphisms (AFLPs), randomly amplified polymorphic DNA (RAPD), sequence-characterized amplified regions (SCARs) and single nucleotide polymorphisms (SNPs) [50]. Nonetheless, microsatellites are still considered as among the most reliable and reproducible interlaboratory techniques across scholars and are also co-dominant markers. Still, despite extreme morphological variations across tomato types, it has been established that the level of genetic polymorphisms detected by genetic markers is rather low [39]. Recently, several studies focusing on simple sequence region germplasm characterization [51,52,53,54] reported a low mean number of alleles across the loci studied ranging from 1.8 [54] to 9.5 [53]. In the current study, 190 genotypes (100 of Cypriot origin) were analyzed and a mean number of 3.2 alleles per locus was established (Table 3). The rather low genetic diversity in the European tomato germplasm seems not to be correlated to the marker system used for fingerprinting. Parisi et al. [44] scored on average 120 SNPs in each tomato chromosome, and less than 20% resulted in polymorphisms. In the current study, the PIC index revealed that on average 0.347 loci are polymorphic across accessions. Recently in Greek tomato landraces [53], a PIC value of approximately 0.7 was reported, while 50% of the SSR loci employed for the characterization of the Bulgarian germplasm were found to be polymorphic [54]. Hence, it can be ascertained that SSRs are a useful marker system for tomato breeding.

Both expected and observed heterozygosity indexes (Table 2) varied but had a generally low range (0.100–0.400 for Ho; 0.098–0.200 for He) indicative of self-pollinated species [55]. Additionally, the majority of Cypriot landraces were found genetically uniform since only one genotype (MLG) was detected, in contrast to Greek heirlooms that presented a more heterogenous composition. This is in accordance with the findings of Terzopoulos and colleagues [39,47] that reported a substantial amount of intra-population heterogeneity and the concurrent presence of several morphotypes within Greek tomato landraces.

All genetic data analyses (hierarchical clustering/dendrogram, MSN, and Bayesian inference) indicated that there is neither a geographical structure nor does clustering correspond with fruit type. This is accordance with several studies that describe a genetic grouping uncorrelated to several morphological traits and fruit characteristics [52,54]. Hence, it can be established that the primer set used in the current study does not correspond to any functional marker linked to phenotypic traits. Moreover, a Mantel test among morphological and genetic distances revealed a significant but rather low-to-moderate correlation. Nonetheless, several significant outcomes could be established that correlate to the agro-morphological analysis. Landraces ARI00735 and ARI00736 were highly affiliated and differed at one locus out of ten hence probably represent different clones of the same landrace. The structure analysis indicated that two major genetic lineages could be distinguished (Figure 5). Most Greek genotypes (from Aegean islands) where clustered in the second genetic group alongside Cypriot landraces ARI00735 and ARI00736. Interestingly, both genotypes from Santorini island (‘Katsari Santorinis’ and ‘Leia Santorinis’) despite having a different fruit type were highly genetically affiliated; hence the possibility of intercrossing among these genotypes cannot be uncritically ruled out. In fact, it has been reported that tomato landraces from Santorini island have a very heterogenous lineage. Tomato farmers in Santorini reserve three discrete types of fruit shape; thus, fulfilling diverse needs: rounded fruits are used for preserves and tomato juice, while slightly flattened or flattened fruits are employed for sun-dried tomatoes [47].

Overall, the current germplasm seems to be genetically distinct and thus could serve as a valuable addition for breeding schemes targeting the genetic enhancement of modern cultivars. Moreover, the employment of heirloom varieties and tomato landraces could reverse the loss of genetic diversity, the farming of allochthonous varieties and ameliorate the ongoing genetic erosion. Moreover, since these landraces have evolved under low input agricultural systems in the semi-arid Cypriot environment, these genotypes might serve as valuable germplasm in sustainable farming systems.

### 4.3. Phytochemical Characterization

Taking into consideration a typical proteinic, lipidic and sugar content in order to describe the nutritional value (in terms of calories), it seems that tomato fruits do not offer a high nutritional value [1]. Still, tomatoes offer an important basis of minerals and nutrients that are defining components for human health promotion, such as antioxidants, ascorbic acid (vitamin C), lycopene and vitamin A (*β*-carotene); Table 5. Thus, tomatoes are the foremost source of lycopene, which presents antioxidant capacity and is assumed to be a protective agent against cardiovascular diseases and malignant tumors [56]. Tomatoes are further considered a vital and outstanding source of ascorbic acid. Comparing with modern cultivars, wild tomato genotypes and landraces are richer in ascorbic acid and may present up to five times more ascorbic acid than their cultivated counterparts [57]. Indeed, Cypriot genotypes can be characterized as highly nutrient tomatoes since they were found to possess significant levels of minerals (21.5 ± 1.38 g/kg DW (N), 5.96 ± 1.35 g/kg DW (P), 2.32 ± 1.00 g/kg DW (Ca), 30.17 ± 5.92 mg/kg DW (Zn), 15.53 ± 3.20 mg/kg DW (Mn) etc.) and vitamins (33.28 ± 8.78 mg/100 g FW vitamin C, 3.55 ± 1.67 mg/100 g FW lycopene and 0.65 ± 0.16 mg/100 g FW *β*-carotene). In that respect, rustic tomato Tuscan varieties were reported to have a three-fold increase in antioxidant phytochemicals, compared to the commercial counterparts [19]. All Cypriot tomato landraces were found to produce fruits of high nutritional value and thus have the potential for commercial exploitation in breeding schemes and trait selection. A clear correlation of fruit shape and phytochemicals was not profound; but in some instances, round tomatoes (landraces ARI00735 and ARI00736) were found to be richer in vitamin C and total phenolics compared to flattened and heart-shaped genotypes. Nonetheless, lycopene levels were somewhat lower than the average. A correlation of fruit type and metabolite levels is not unprecedented, and has been described previously. Carli and coworkers [58,59] and Figas et al. [60] reported that round and elongated fruit tomato types have higher levels of sugars and dry weight compared to other fruit types studied. Recently, a close affiliation of alkaloids to specific fruit types was also reported [61]. Moreover, the authors suggested that high levels of these metabolites could serve as a dual-purpose element for pathogen resilience, as well as flavor enhancement and thus may present a desirable future breeding objective.

Cypriot tomato fruits also contained significant amounts of phenolic compounds (averaging 7 mg GAE/100 g FW). It has been reported that phenolics are a highly desirable feature since these moieties encompass the major contribution of antioxidant activities in tomato fruits [62]. Flattened and heart-shaped tomato fruits were found to be rather homogeneous for total phenolics but landrace ARI00734 (the only one having a high-rounded type) presented substantially lower levels (Table 5). A superiority of flattened tomato fruits in phenolic concentration has also been reported [61]. Interestingly, the levels of total phenolics naturally present in the Cypriot tomato collection are comparable to the ones reported in greenhouse-cultivated tomato fruits (4.3 to 8.5 GAE/100 g FW) after the addition of biofertilizer (*Bacillus licheniformis*) on a commercial cultivar [36]. Nonetheless, high levels of phenolics are not a strictly genotypic regulated trait, but are rather the outcome of a genotype X environment interaction. Stressor factors that can induce ROS in tissues have been implicated in the increase of phenolics, in order to ameliorate cellular damage [63].

A critical element of consumers’ demand for tomato fruits is the TSS and acidity characteristics. The pH parameter ranged from 4.55 (ARI00732) to 4.68 (ARI00735); hence Cypriot tomatoes do not present an elevated acidity. Scarano and colleagues analyzed the phytochemical composition of an Italian landrace tomato germplasm collection under an elevated temperature and established a very narrow pH range (4.3 to 4.6) that corresponds to the present study [64]. Nonetheless, the TSS content of the Cypriot landraces has an overall lower value (4.11 °Brix on average and a maximum of 5 °Brix) compared to other local germplasms where values larger than 5 °Brix are reported [42,45,64]. The sugar content is heavily influenced by both temperature and light spectra; hence the different environmental conditions and cultivation practices across diverse studies can hamper direct comparisons among landraces grown at distant locations and timelines. Moreover, postharvest treatments can severely distort TSS levels. Kasim and Kasim reported that the fructose, glucose, and the TSS content of tomatoes treated with UVB light at the red ripe stage were found to be positively affected [65]. Moreover, earlier studies reported that TSS content for large beefsteak tomatoes fluctuated from 3 to 5 °Brix, for medium-sized tomato fruits values ranged from 5 to 7%, and for small cherry tomatoes the TSS content was estimated to be above 9% [66,67]. As a result, it can be established that the fruit size is negatively correlated to sugar content. In the current study, Cypriot landraces were found to produce rather large fruits; hence the relatively low sugar levels may be attributed to augmented size.

## 5. Conclusions

The present study provides the first attempt to characterize and evaluate the traditional Cypriot tomato landraces. For that purpose, a multidisciplinary combined experimental approach was adopted. Twenty-four IPGRI descriptors were used in order to identify morphological and agronomical affinities, and it was determined that morphotypes were largely grouped according to fruit-type (flattened, heart-shaped, rounded and highly rounded accessions). Cypriot and foreign tomato germplasm was also genotyped by means of a single-tube microsatellite analysis and it was established that a rather limited amount of intra-variability exists within landraces. Nonetheless, it was ascertained that complex interchange of genetic material must have taken place in the tomato lineages. Finally, mineral levels and bioactive phytochemicals were determined in order to acquire the nutrient profile of these traditional tomato types and their eligibility in future breeding efforts.

## Figures and Tables

**Table 1:** Tomato genotypes used in the current study.

No	Code	Origin	Fruit Shape	Fruit Size
1.	AR100731	Cypriot	Flattened	Very large
2.	ARI00732	Cypriot	Flattened	Very large
3.	ARI00733	Cypriot	Heart-shaped	Large
4.	ARI00734	Cypriot	High rounded	Small
5.	ARI00735	Cypriot	Rounded	Medium
6.	ARI00736	Cypriot	Rounded	Medium
7.	ARI00737	Cypriot	Flattened	Very large
8.	ARI00872	Cypriot	Flattened	Very large
9.	ARI00905	Cypriot	Heart-shaped	Large
10.	ARI00906	Cypriot	Heart-shaped	Large
11.	‘Marmande’	French	Rounded	Medium
12.	‘Kardoula’	Greek	Heart-shaped	Medium
13.	‘Katsari Santorinis’	Greek	Rounded	Small
14.	‘Konti Kithiron’	Greek	Elliptical shaped	Medium
15.	‘Leia Santorinis’	Greek	Rounded	Small
16.	‘Megali Stroggili’	Greek	Rounded	Large
17.	‘Stithos Aphroditis’	Greek	Elongated	Medium
18.	‘Chiou’	Greek	Rounded	Small
19.	‘Chondrokatsari’	Greek	Flattened	Large

**Table 2:** Genetic diversity of landraces and heirloom varieties across the analyzed loci.

Genotypes	N	Na	Ne	I	Ho	He	uHe	F	MLGs
ARI00731	10	1.400	1.400	0.277	0.400	0.200	0.211	−1.000	1
ARI00732	10	1.300	1.300	0.208	0.300	0.150	0.158	−1.000	1
ARI00733	10	1.200	1.200	0.139	0.200	0.100	0.105	−1.000	1
ARI00734	10	1.200	1.200	0.139	0.200	0.100	0.105	−1.000	1
ARI00735	10	1.300	1.210	0.158	0.210	0.110	0.115	−0.684	2
ARI00736	10	1.400	1.320	0.240	0.290	0.168	0.176	−0.455	2
ARI00737	10	1.300	1.300	0.208	0.300	0.150	0.158	−1.000	1
ARI00872	10	1.300	1.300	0.208	0.300	0.150	0.158	−1.000	1
ARI00905	10	1.300	1.300	0.208	0.300	0.150	0.158	−1.000	1
ARI00906	10	1.200	1.200	0.139	0.200	0.100	0.105	−1.000	1
‘Marmande’	10	1.400	1.345	0.261	0.200	0.184	0.194	0.000	2
‘Kardoula’	10	1.200	1.192	0.137	0.180	0.098	0.103	−0.833	2
‘Katsari Santorinis’	10	1.400	1.242	0.187	0.200	0.123	0.129	−0.278	3
‘Konti Kithiron’	10	1.500	1.281	0.235	0.230	0.157	0.165	−0.274	4
‘Leia Santorinis’	10	1.500	1.422	0.310	0.220	0.218	0.229	−0.022	3
‘Megali Stroggili’	10	1.500	1.332	0.269	0.240	0.178	0.187	−0.217	3
‘Stithos Aphroditis’	10	1.100	1.100	0.069	0.100	0.050	0.053	−1.000	1
‘Chiou’	10	1.300	1.265	0.198	0.180	0.140	0.147	−0.222	3
‘Chondrokatsari’	10	1.300	1.222	0.171	0.200	0.118	0.124	−0.333	2
Average	10	1.321	1.270	0.198	0.234	0.139	0.146	−0.648	33 (Total)

**Table 3:** Allele summary statistics and diversity indexes across the analyzed loci.

Locus	Number of Alleles	PIC	Dp	Hs	Ht	Gst	Gprime_st	D
EST253712	3	0.346	0.889	0.023	0.302	0.923	0.949	0.301
EST258529	4	0.372	0.803	0.414	0.593	0.301	0.534	0.322
LE20592	3	0.352	0.877	0.055	0.193	0.712	0.766	0.153
LELEUZIP	4	0.375	0.753	0.528	0.598	0.117	0.260	0.156
LEMDDNA	3	0.374	0.749	0.290	0.590	0.509	0.735	0.446
LESSRPSPGb	3	0.347	0.888	0.048	0.640	0.924	0.975	0.657
TMS42	3	0.346	0.856	0.005	0.409	0.987	0.993	0.429
TMS52	4	0.310	0.937	0.005	0.732	0.993	0.998	0.771
TMS58	2	0.294	0.724	0.018	0.081	0.780	0.804	0.068
TMS59	3	0.351	0.889	0.076	0.322	0.763	0.836	0.281
Average	3.2	0.347	0.837	0.146	0.446	0.672	0.801	0.370

**Table 4:** Mineral composition across tomato genotypes.

Landrace	N (g/kg)	P (g/kg)	Ca (g/kg)	Mg (g/kg)	K (g/kg)	Na (g/kg)	Zn (mg/kg)	Mn (mg/kg)	Cu (mg/kg)
AR100731	21.0 ± 0.27 ^bc^	7.12 ± 0.11 ^b^	2.64 ± 0.09 ^b^	1.66 ± 0.02 ^b^	36.32 ± 3.54 ^b^	1.34 ± 0.01 ^de^	23.14 ± 1.60 ^gh^	17.22 ± 0.25 ^b^	8.61 ± 0.18 ^de^
ARI00732	20.2 ± 0.48 ^c^	4.90 ± 0.04 ^f^	1.38 ± 0.16 ^e^	1.60 ± 0.01 ^bc^	31.93 ± 0.98 ^cd^	1.39 ± 0.00 ^c^	29.62 ± 0.94 ^de^	13.50 ± 0.51 ^bc^	6.55 ± 0.45 ^ef^
ARI00733	18.6 ± 0.32 ^d^	3.61 ± 0.05 ^g^	1.64 ± 0.05 ^de^	1.43 ± 0.02 ^d^	31.86 ± 0.23 ^cd^	1.12 ± 0.00 ^g^	22.08 ± 1.81 ^h^	12.53 ± 0.55 ^c^	9.63 ± 1.32 ^d^
ARI00734	21.6 ± 0.22 ^b^	5.49 ± 0.09 ^d^	2.19 ± 0.14 ^c^	1.46 ± 0.03 ^d^	32.50 ± 0.45 ^bd^	1.20 ± 0.01 ^f^	30.53 ± 0.54 ^cd^	14.08 ± 1.48 ^bc^	12.40 ± 0.95 ^c^
ARI00735	21.3 ± 0.17 ^b^	7.22 ± 0.03 ^b^	4.24 ± 0.02 ^a^	1.65 ± 0.05 ^bc^	32.17 ± 0.03 ^bd^	1.37 ± 0.01 ^cd^	36.54 ± 0.45 ^b^	22.56 ± 1.53 ^a^	16.76 ± 1.25 ^b^
ARI00736	20.2 ± 0.28 ^c^	8.00 ± 0.08 ^a^	3.96 ± 0.13 ^a^	2.08 ± 0.03 ^a^	43.23 ± 0.26 ^a^	1.33 ± 0.03 ^e^	41.65 ± 0.35 ^a^	17.21 ± 1.06 ^b^	19.83 ± 0.30 ^a^
ARI00737	22.0 ± 0.069 ^b^	6.49 ± 0.13 ^c^	1.85 ± 0.03 ^d^	1.65 ± 0.01 ^bc^	33.21 ± 1.69 ^bd^	1.33 ± 0.01 ^e^	25.85 ± 1.53 ^fg^	17.28 ± 1.15 ^b^	18.12 ± 0.13 ^ab^
ARI00872	21.0 ± 0.14 ^bc^	6.61 ± 0.08 ^c^	2.24 ± 0.01 ^c^	1.56 ± 0.01 ^c^	34.82 ± 0.08 ^bc^	1.36 ± 0.01 ^ce^	27.25 ± 0.35 ^ef^	12.18 ± 2.06 ^c^	10.20 ± 0.12 ^cd^
ARI00905	21.0 ± 0.15 ^bc^	4.89 ± 0.03 ^f^	1.72 ± 0.06 ^d^	1.56 ± 0.06 ^c^	32.18 ± 0.21 ^bd^	1.68 ± 0.03 ^a^	33.25 ± 1.20 ^c^	14.52 ± 0.53 ^bc^	5.91 ± 0.78 ^f^
ARI00906	23.6 ± 0.55 ^a^	5.25 ± 0.07 ^e^	1.34 ± 0.07 ^e^	1.63 ± 0.02 ^bc^	29.73 ± 0.82 ^d^	1.57 ± 0.01 ^b^	31.87 ± 1.36 ^cd^	14.42 ± 1.13 ^bc^	8.65 ± 0.25 ^de^
Average	21.5 ± 1.38	5.96 ± 1.35	2.32 ± 1.00	1.63 ± 0.18	33.79 ± 3.90	1.37 ± 0.16	30.17 ± 5.92	15.53 ± 3.20	11.66 ± 4.81
CV%	6.55	22.59	43.13	10.76	11.57	11.48	19.75	20.79	41.40
ANOVA *p* value	*p* = 8 × 10^−8^	*p* = 5.8 × 10^−10^	*p* = 5.2 × 10^−9^	*p* = 1.8 × 10^−6^	*p* = 0.0015	*p* = 1.2 × 10^−9^	*p* = 4 × 10^−7^	*p* = 0.0011	*p* = 4.5 × 10^−7^

Mean values and relative standard deviations across mineral analyses. Different letters (a–g) refer to statistically significant differences at *p* < 0.05 as analyzed by one-way ANOVA and the Duncan Multiple Range Test. Coefficient of variation (CV) was calculated as the ratio of the standard deviation to mean values.

**Table 5:** Assessment of tomato fruit quality traits for Cypriot landraces analyzed.

Landrace	pH	TA (g/L)	TSS (°Brix)	TSS/TA	Vitamin C (mg/100 g FW)	TP (mg GAE/100 g FW)	Lycopene (mg/100 g FW)	*β*-Carotene (mg/100 g FW)
AR100731	4.56 ± 0.02 ^d^	3.04 ± 0.05 ^d^	3.80 ± 0.10 ^d^	12.48 ± 0.25 ^bc^	39.60 ± 1.55 ^b^	7.95 ± 0.64 ^ab^	4.37 ± 0.19 ^d^	0.68 ± 0.04 ^bcd^
ARI00732	4.55 ± 0.02 ^d^	3.90 ± 0.03 ^a^	5.07 ± 0.06 ^ab^	12.83 ± 0.12 ^b^	24.40 ± 2.98 ^d^	5.67 ± 0.22 ^c^	5.47 ± 0.03 ^b^	0.65 ± 0.03 ^bcd^
ARI00733	4.59 ± 0.01 ^cd^	3.63 ± 0.07 ^b^	4.80 ± 0.06 ^ab^	13.22 ± 0.37 ^b^	30.80 ± 0.19 ^c^	5.36 ± 0.31 ^cd^	4.52 ± 0.03 ^cd^	0.42 ± 0.07 ^e^
ARI00734	4.66 ± 0.02 ^ab^	3.06 ± 0.05 ^d^	3.33 ± 0.07 ^ef^	10.89 ± 0.13 ^d^	30.00 ± 3.22 ^c^	4.50 ± 0.16 ^d^	1.42 ± 0.05 ^g^	0.54 ± 0.04 ^ce^
ARI00735	4.68 ± 0.01 ^a^	3.05 ± 0.06 ^d^	3.20 ± 0.15 ^f^	10.49 ± 0.35 ^d^	48.02 ± 0.94 ^a^	8.87 ± 0.29 ^a^	1.49 ± 0.07 ^g^	0.92 ± 0.02 ^a^
ARI00736	4.63 ± 0.03 ^ac^	3.12 ± 0.03 ^d^	3.53 ± 0.07 ^de^	11.33 ± 0.28 ^cd^	47.30 ± 1.65 ^a^	7.21 ± 0.21 ^b^	1.55 ± 0.05 ^g^	0.79 ± 0.10 ^ab^
ARI00737	4.60 ± 0.01 ^bcd^	3.31 ± 0.04 ^c^	5.07 ± 0.18 ^a^	15.29 ± 0.35 ^a^	31.12 ± 0.41 ^c^	7.78 ± 0.57 ^ab^	4.84 ± 0.10 ^c^	0.69 ± 0.04 ^bc^
ARI00872	4.67 ± 0.01 ^a^	2.85 ± 0.05 ^e^	3.23 ± 0.06 ^f^	11.32 ± 0.16 ^cd^	31.45 ± 0.23 ^c^	7.33 ± 0.34 ^b^	2.22 ± 0.24 ^f^	0.63 ± 0.03 ^bcd^
ARI00905	4.59 ± 0.01 ^cd^	3.32 ± 0.03 ^c^	4.37 ± 0.03 ^c^	13.16 ± 0.02 ^b^	26.17 ± 0.76 ^cd^	7.91 ± 0.20 ^ab^	5.85 ± 0.04 ^a^	0.73 ± 0.02 ^b^
ARI00906	4.56 ± 0.03 ^d^	3.70 ± 0.02 ^b^	4.73 ± 0.04 ^b^	12.81 ± 0.17 ^b^	24.37 ± 1.40 ^d^	6.93 ± 0.13 ^b^	3.83 ± 0.11 ^e^	0.49 ± 0.11 ^de^
Average	4.61 ± 0.05	3.30 ± 0.33	4.11 ± 0.75	12.40 ± 1.43	33.28 ± 8.78	6.95 ± 1.40	3.55 ± 1.67	0.65 ± 0.16
CV%	0.99	9.83	17.89	10.89	24.88	18.58	46.11	21.21
ANOVA *p* value	*p* = 0.00018	*p* = 2.2 × 10^−12^	*p* = 5.3 × 10^−13^	*p* = 4.5 × 10^−10^	*p* = 2.5 × 10^−09^	*p* = 3.7 × 10^−7^	*p* = 2.2 × 10^−16^	*p* = 0.00037

Mean values and relative standard deviations across phytochemical analyses. Different letters (a–g) refer to statistically significant differences at *p* < 0.05 as analyzed by one-way ANOVA and the Duncan Multiple Range Test. Coefficient of variation (CV) was calculated as the ratio of the standard deviation to mean values.

## Supplementary Materials

The following are available online at https://www.mdpi.com/article/10.3390/plants10081698/s1, Figure S1: Quantile-quantile (QQ) plot of residuals. Grey area represents the 95% confidence interval (A). Distribution of the number of locules across Cypriot tomato landraces (B). Figure S2: Correlation and hierarchical clustering of agro-morphological traits as established for quantitative traits. Figure S3: Levels of attributes explaining variation across Cypriot tomato landraces. Figure S4: Single run electropherogram setup depicting the concurrent analysis of the ten loci employed. Figure S5: Genotype accumulation curve for Cypriot accessions. Proportion of MLGs identified based on the number sampled loci and 1000 randomizations. Figure S6: Estimates of genotypes differentiation based on population diversity indexes. Figure S7: Probability of Hardy Weinberg disequilibrium for the loci studied depicted as a heatmap. Table S1: IPGRI descriptors. Table S2: Primers used. Table S3: Morphological characters. Table S4: *F_st_* values.

## Abbreviations

2:6-dichloroindophenol sodium salt: DCIP; Agricultural Research Institute: ARI; Analysis of Molecular Variance: Amova; approximate Likelihood-Ratio Test: aLRT; Ascorbic Acid: AA; Coefficient of Variation: CV; Crop Wild Relatives: CWRs; Discriminant Analysis of Principal Components: DAPC; Dry Weight: DW; Fresh Weight: FW; Hardy–Weinberg Equilibrium: HWE; International Plant Genetic Resources Institute: IPGRI; Minimum Spanning Network: MSN; Multi-Locus Genotype: MLG; Principal Component Analysis: PCA; Reactive Oxygen Species: ROS; Simple Sequence Repeats: SSRs; Soluble Solid Content: SSC; Titratable Acidity: TA; Total Phenols: TP; Total Soluble Solids: TSS.
