# Supplementary figures and images for: Morphological Diversity, Genetic Characterization, and Phytochemical Assessment of the Cypriot Tomato Germplasm
Source: Plants (Basel). 2021 Aug 18;10(8):1698. doi: 10.3390/plants10081698 (PMC8401825; doi:10.3390/plants10081698)

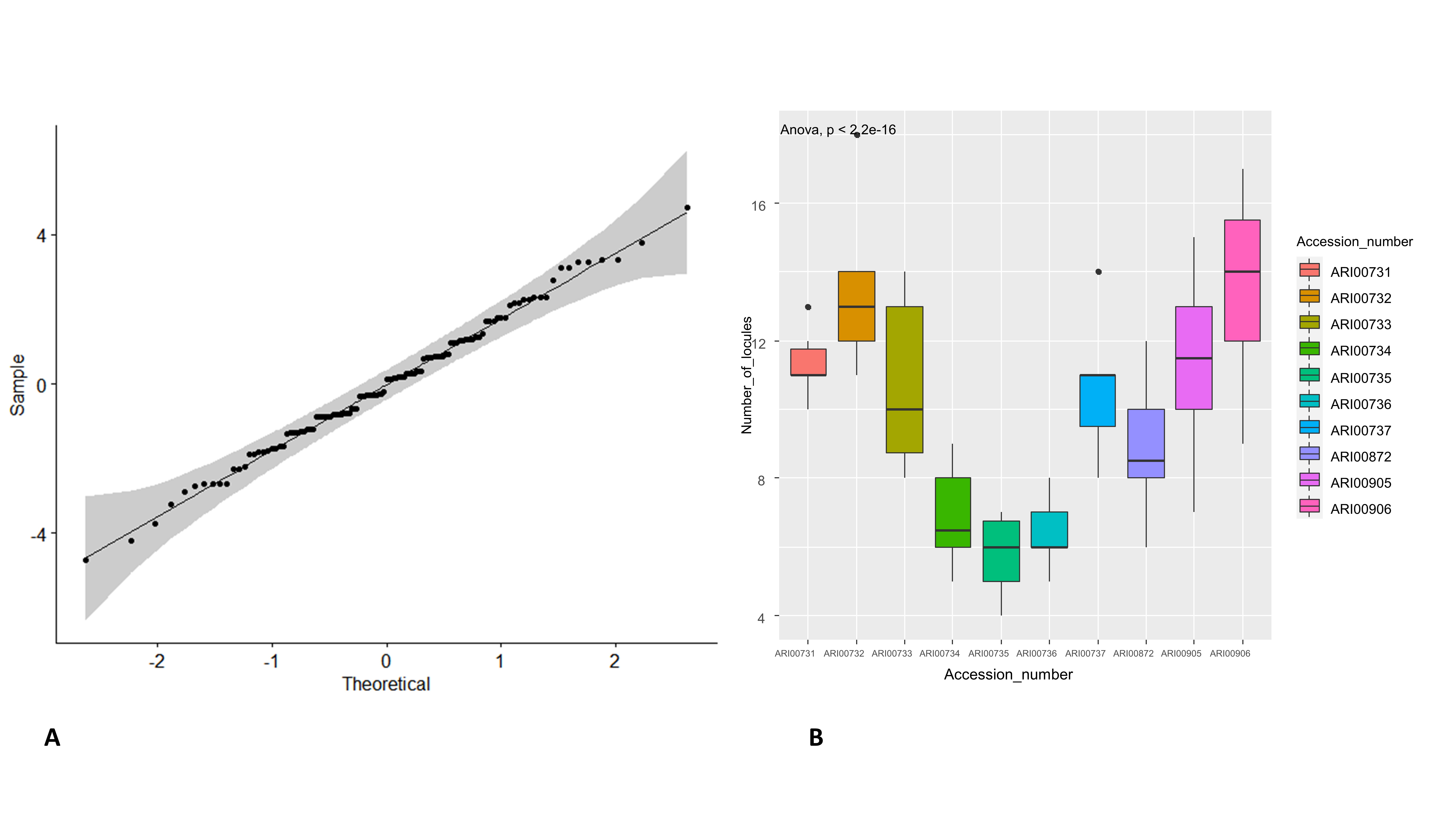

Supplement: Supplementary file 1 [file plants-10-01698-s001.zip › supl figure 1.jpg]

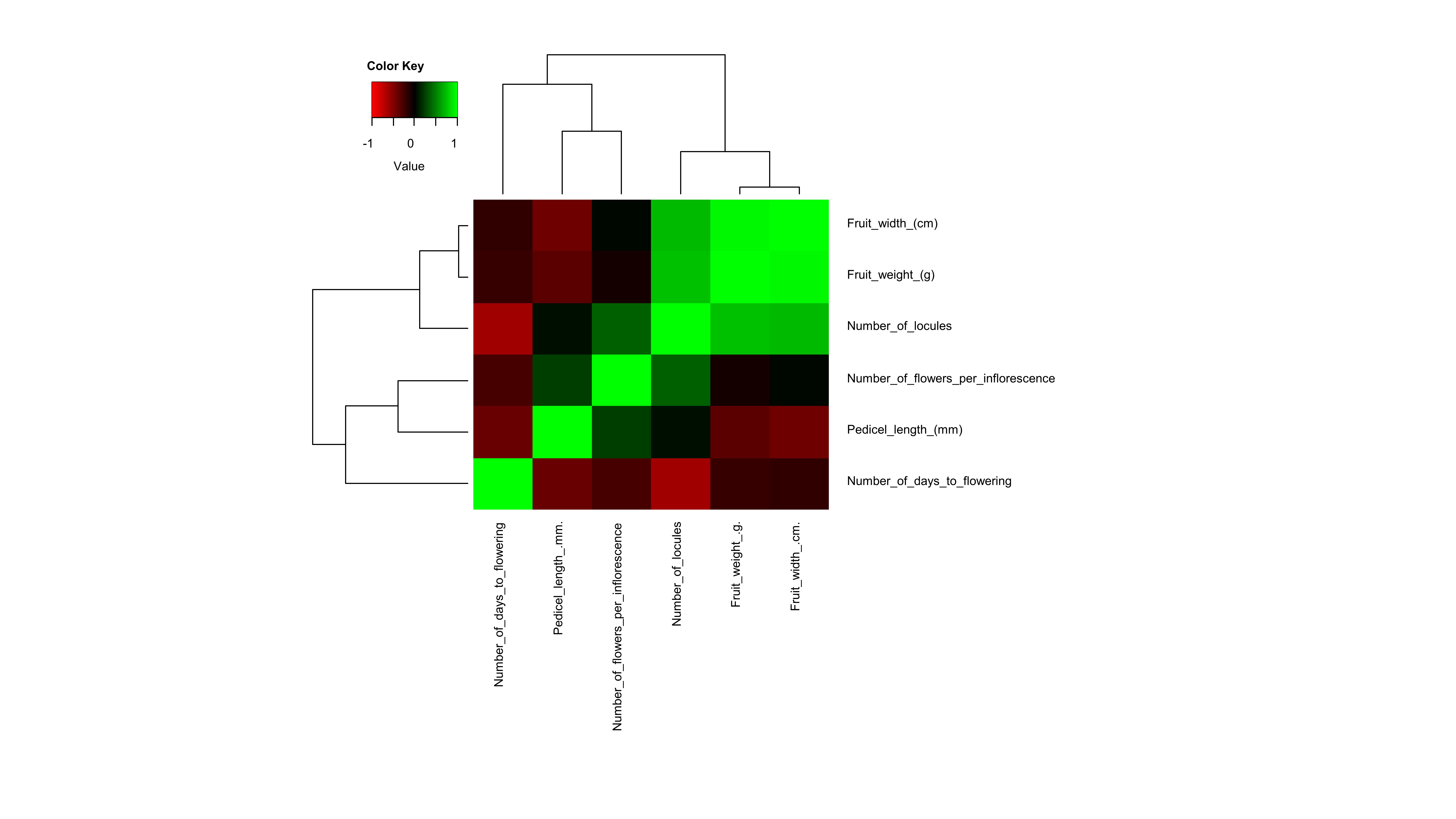

Supplement: Supplementary file 1 [file plants-10-01698-s001.zip › supl figure 2.jpg]

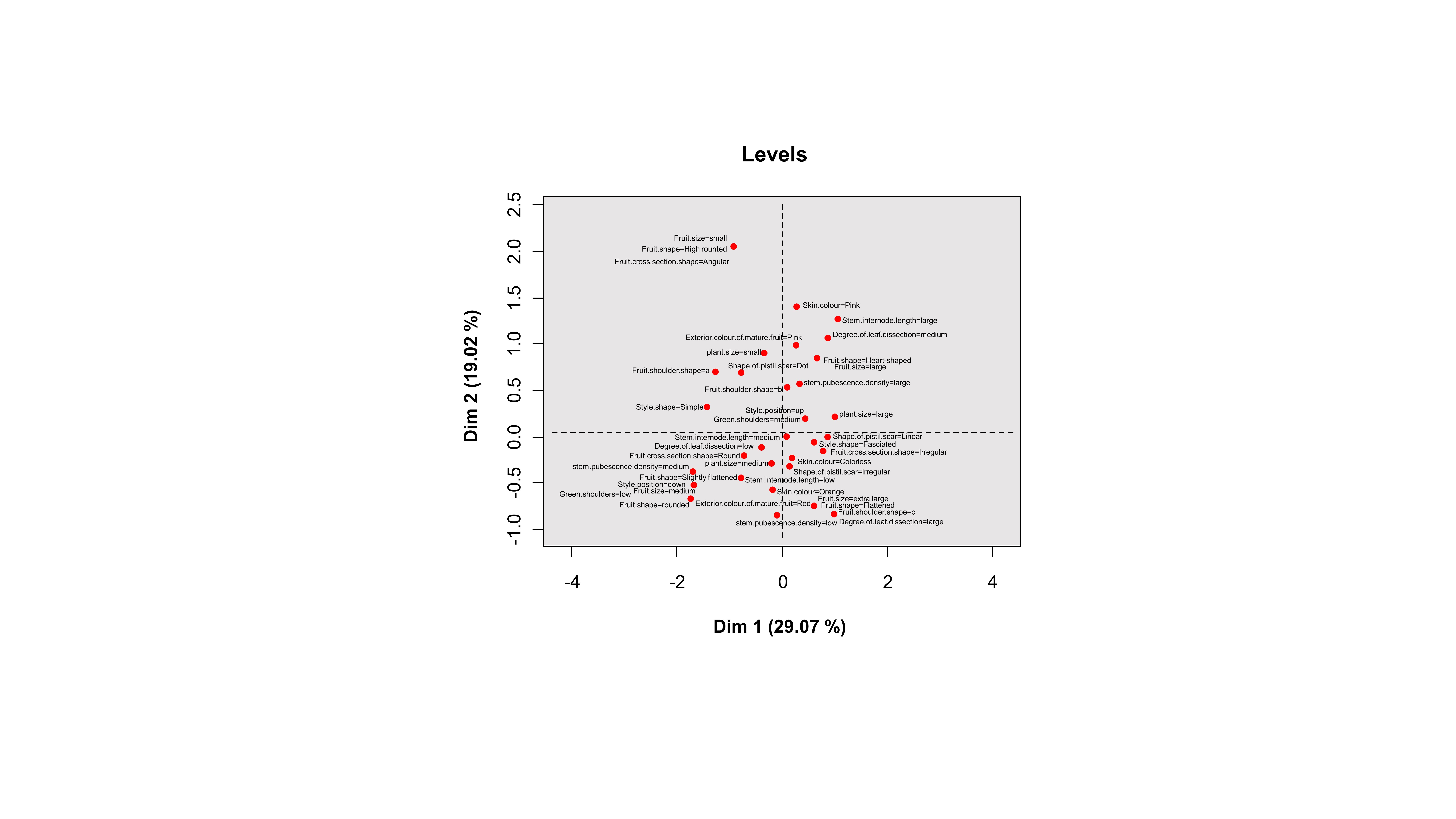

Supplement: Supplementary file 1 [file plants-10-01698-s001.zip › supl figure 3.jpg]

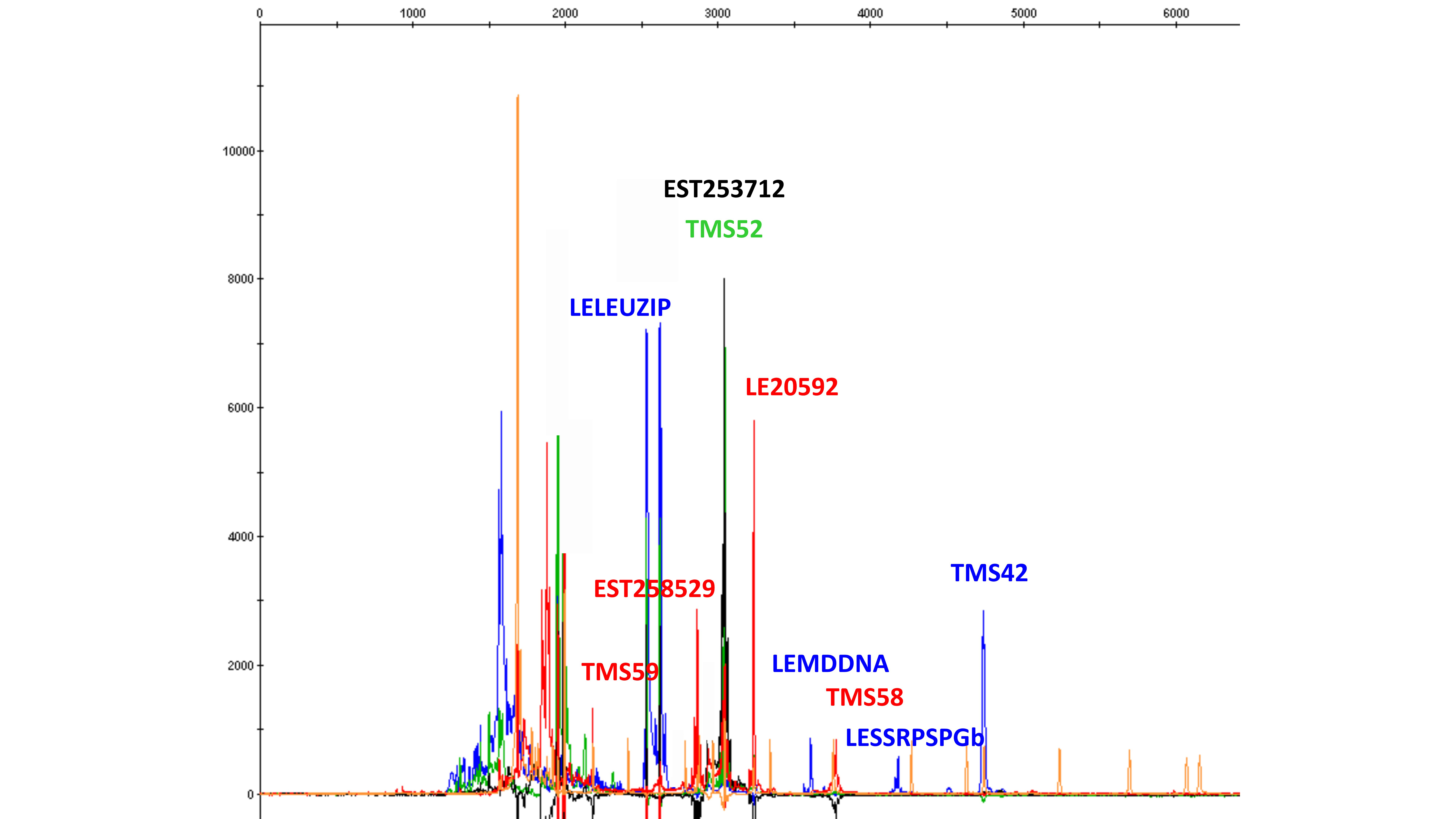

Supplement: Supplementary file 1 [file plants-10-01698-s001.zip › supl figure 4.jpg]

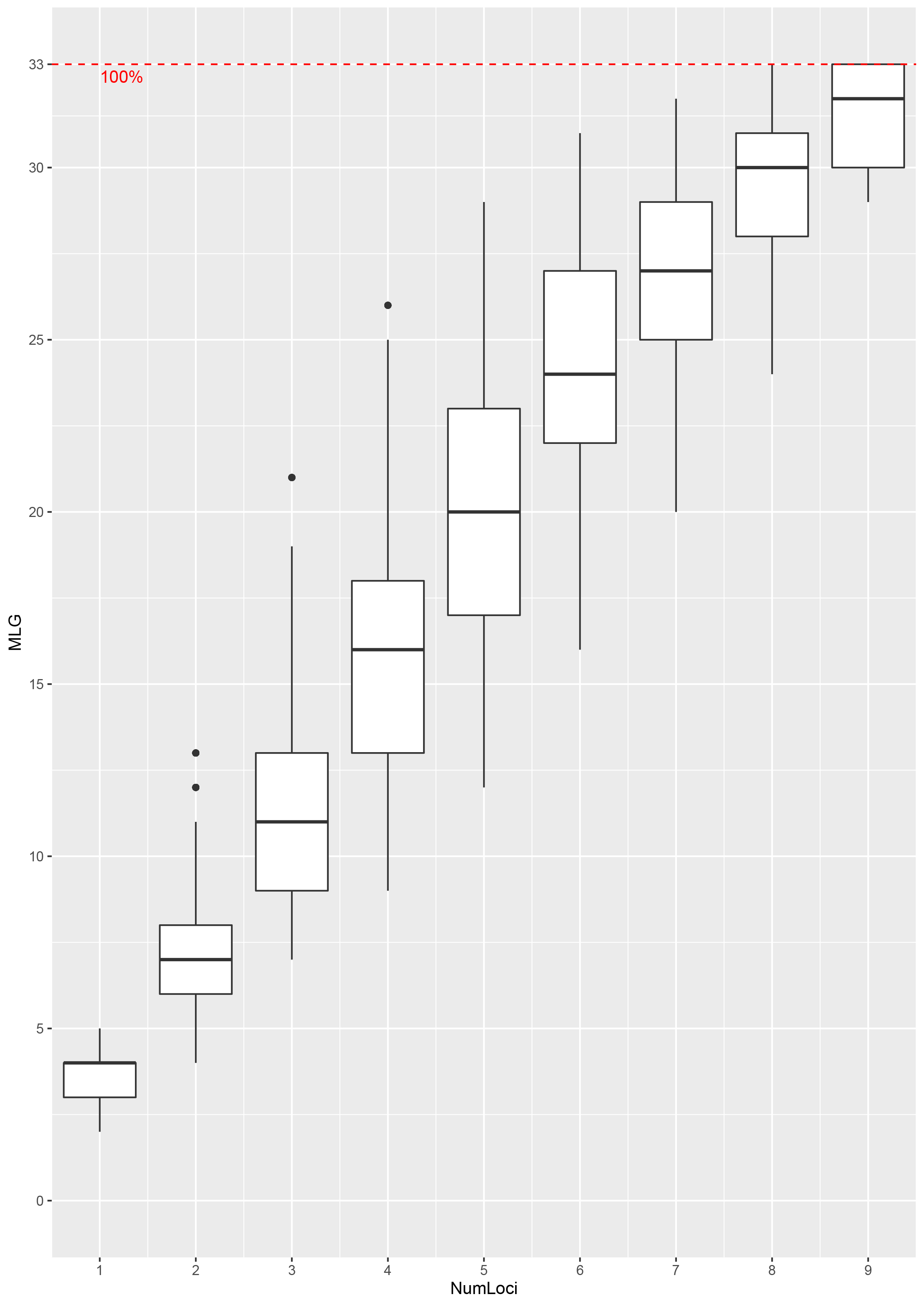

Supplement: Supplementary file 1 [file plants-10-01698-s001.zip › supl figure 5.jpg]

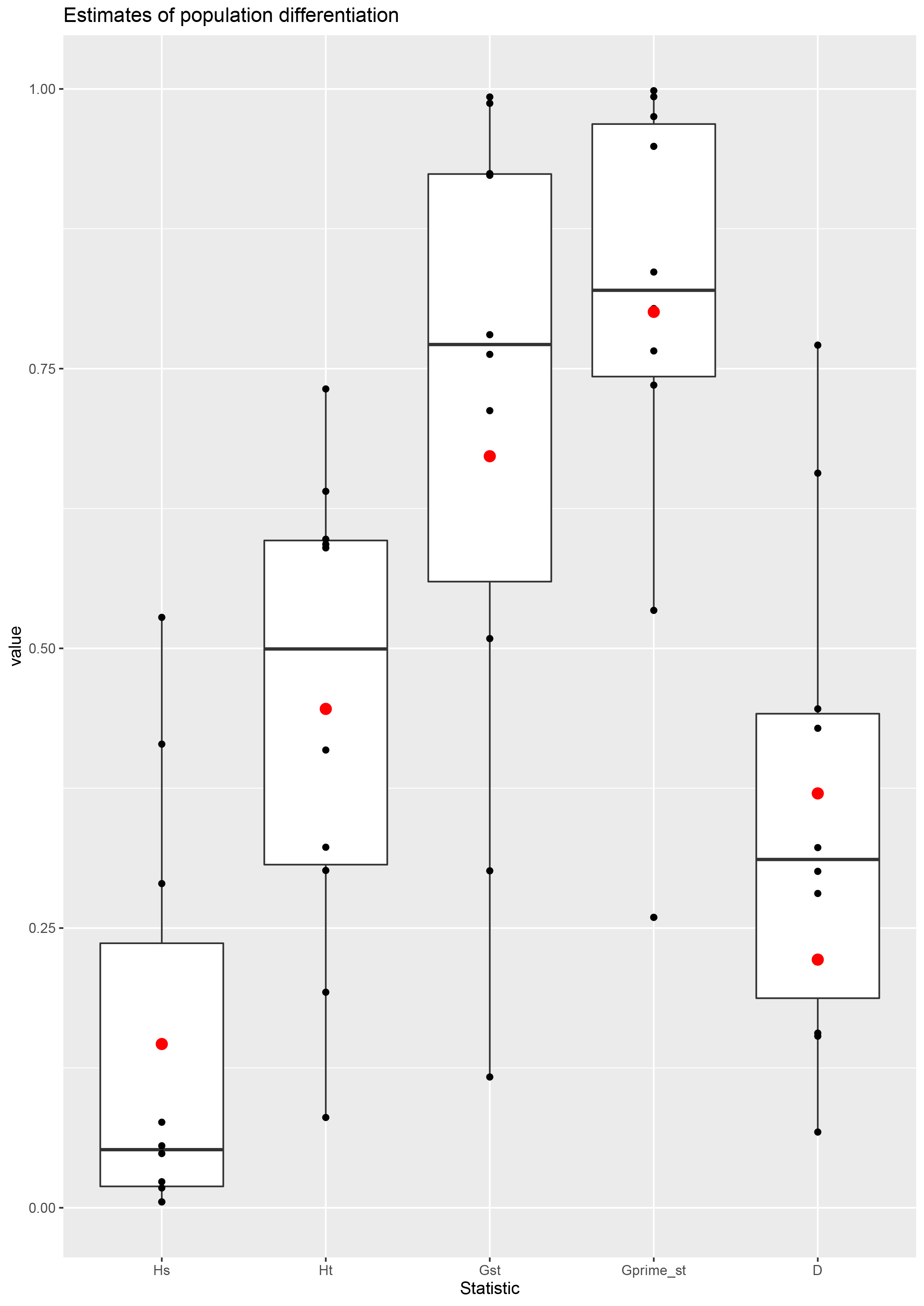

Supplement: Supplementary file 1 [file plants-10-01698-s001.zip › supl figure 6.jpg]

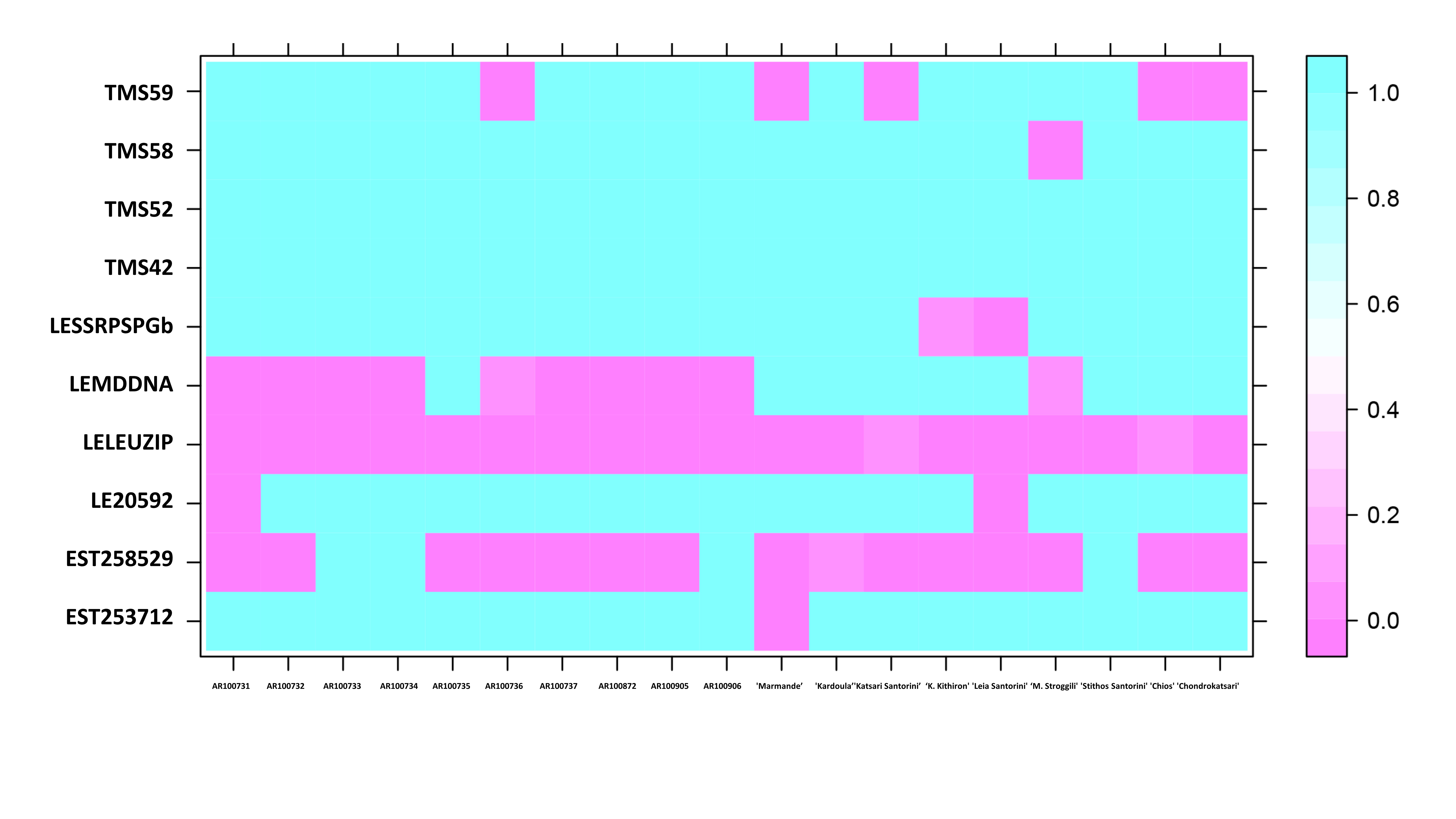

Supplement: Supplementary file 1 [file plants-10-01698-s001.zip › supl figure 7.jpg]
